# Longevity-Related Gene Transcriptomic Signature in Glioblastoma Multiforme

**DOI:** 10.1155/2018/8753063

**Published:** 2018-03-21

**Authors:** Manal S. Fawzy, Dahlia I. Badran, Essam Al Ageeli, Saeed Awad M. Al-Qahtani, Saleh Ali Alghamdi, Ghada M. Helal, Eman A. Toraih

**Affiliations:** ^1^Department of Medical Biochemistry, Faculty of Medicine, Suez Canal University, Ismailia 41522, Egypt; ^2^Department of Medical Biochemistry, Faculty of Medicine, Northern Border University, Arar, Saudi Arabia; ^3^Center of excellence in Molecular and Cellular Medicine, Faculty of Medicine, Suez Canal University, Ismailia, Egypt; ^4^Department of Clinical Biochemistry (Medical Genetics), Faculty of Medicine, Jazan University, Jazan, Saudi Arabia; ^5^Department of Physiology, Faculty of Medicine, Taibah University, Almadinah Almunawwarah, Saudi Arabia; ^6^Medical Genetics, Clinical Laboratory Department, College of Applied Medical Sciences, Taif University, Taif, Saudi Arabia; ^7^Department of Medical Biochemistry, Faculty of Medicine, Mansoura University, Mansoura, Egypt; ^8^Genetics Unit, Histology and Cell Biology Department, Faculty of Medicine, Suez Canal University, Ismailia, Egypt

## Abstract

Glioblastoma multiforme (GBM) (grade IV astrocytoma) has been assumed to be the most fatal type of glioma with low survival and high recurrence rates, even after prompt surgical removal and aggressive courses of treatment. Transcriptional reprogramming to stem cell-like state could explain some of the deregulated molecular signatures in GBM disease. The present study aimed to quantify the expression profiling of longevity-related transcriptional factors *SOX2*, *OCT3/4*, and *NANOG* to evaluate their diagnostic and performance values in high-grade gliomas. Forty-four specimens were obtained from glioblastoma patients (10 females and 34 males). Quantitative real-time polymerase chain reaction was applied for relative gene expression quantification. In silico network analysis was executed. *NANOG* and *OCT3/4* mRNA expression levels were significantly downregulated while that of *SOX2* was upregulated in cancer compared to noncancer tissues. Receiver operating characteristic curve analysis showed high diagnostic performance of *NANOG* and *OCT3/4* than *SOX2*. However, the aberrant expressions of the genes studied were not associated with the prognostic variables in the current population. In conclusion, the current study highlighted the aberrant expression of certain longevity-associated transcription factors in glioblastoma multiforme which may direct the attention towards new strategies in the treatment of such lethal disease.

## 1. Introduction

Tumors of the brain were considered one of the ten most common causes of cancer-related mortality [1]. According to the World Health Organization (WHO) classification, the primary brain tumors are categorized into glial tumors (e.g., glioblastoma, astrocytomas, oligodendroglial tumors, and ependymal tumors), embryonic tumors (e.g., medulloblastomas), tumors of the meninges, tumors of the hematopoietic system, and tumors of the sellar region [2]. The most fatal type of glioma has been reported to be the glioblastoma multiforme (GBM) [3] which represents up to 50% of almost all primary brain gliomas [4] with poor prognosis [5] and median survival rate of nearly 25 months after treatment [6]. The recurrence of the tumor after prompt surgical removal despite the aggressive courses of radio- and chemotherapy denotes the limited understanding of the disease biology [7]. Dell'Albani has stated that “new insights into the causes and the potential treatment of CNS tumors have come from disclosing relations with genes that regulate cell growth, proliferation, differentiation, and death during normal development” [7]. These genes may represent a new target for GBM treatment by ameliorating the survival rate and preventing or minimizing disease recurrence.

Several emerging evidences support the reactivation of pluripotent transcription factors in many types of cancer [8–12]. As a normal biological phenomenon, these factors are expressed in embryonic stem cells (ESCs) and somatic cells where they imply the self-renewal [13] and the pluripotency characteristics [14]. As cancer development is a multistep process in which differentiated cells transform into immature ones, these factors could participate in cancer biogenesis and/or progress.

Among these pluripotent transcription factors overexpressed in high-grade gliomas are “sex-determining region Y-Box (SOX2), octamer-binding transcription factor 4 (OCT 4), and Nanog homeobox (NANOG)” [13, 15].

**Figure 1:**
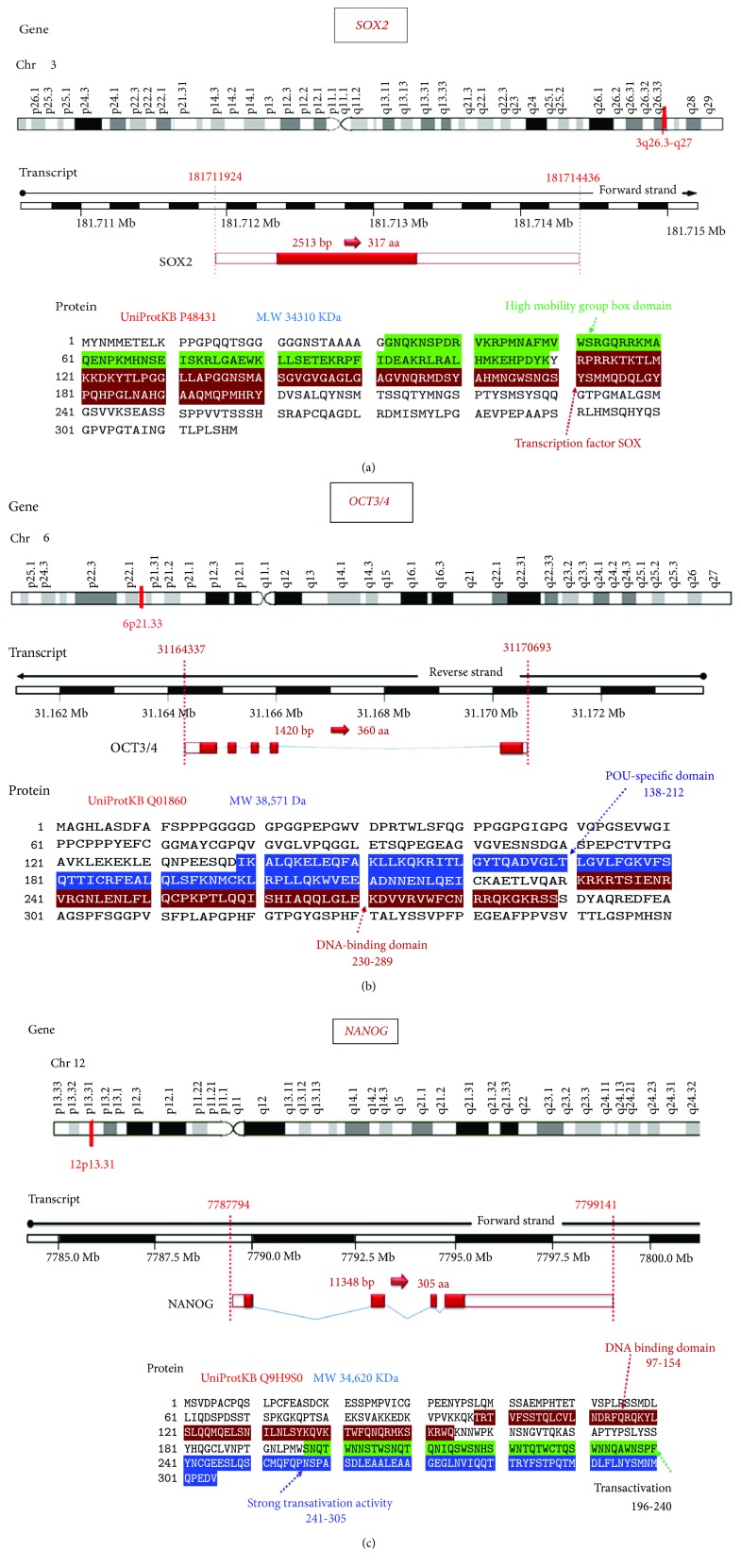
Structural analysis of the studied longevity-related genes. (a) *SOX2* gene (OMIM 184429) location in chromosome 3q26.33. The complete gene spans 2513 bases of genomic DNA (NC_000003.12; Chr 3: 181,711,924 to 181,714,436, plus strand; human genome assembly GRCh38). This intronless gene encodes a member of the SRY-related HMG-box family of transcription factors (translation length: 317 amino acids). *SOX2* refers to the primary and predominant transcript of the *SOX2* gene. The high-mobility group box domain amino acid sequence in the SOX2 transcription factor is highlighted by green color and the DNA-binding domain sequences are brown colored. (b) *NANOG* gene (OMIM: 607937) location in chromosome 12p13.31. The complete gene spans 11,348 bases of genomic DNA (NC_000012.12; Chr 12: 7,787,794 to 7,799,141, plus strand). The encoded protein (305 amino acids). (c) *OCT3/4* gene (OMIM: 164177) location in chromosome 6p21.33. The gene spans 1420 bases of genomic DNA (NC_00006.12; genomic coordinates (GRCh38): 6: 31,164,337-31,170,693, minus strand). The gene encodes a transcription factor (360 amino acids) containing a POU-specific homeodomain (blue amino acid sequences) and DNA-binding domain (brown amino acid sequences). MW: molecular weight; Da: Dalton; aa: amino acids (data source: http://Genecards.org, http://Ensembl.org and UniProtKB).

*SOX2* gene encodes a transcriptional factor (TF) of 317 amino acids which contains a high-mobility group DNA-binding domain (Figure 1(a)) [16]. It implicated in embryonic development regulation, cell fate determination, and embryonic stem cell pluripotency. More specifically, it was reported to control the neural stem cell proliferation and differentiation into neurons, astrocytes, or oligodendrocytes [17]. *SOX2* is expressed in stem cells of endoderm-derived organs such as the liver, pancreas, and stomach [18], and its aberrant expression has been found to support self-renewal and inhibit neuronal differentiation [19]. Additionally, *SOX2* knockout in glioblastoma stem cells isolated from human glioma tumor inhibits cell proliferation and tumorigenicity in immunodeficient mice [20].

OCT3/4 is a member of a transcription POU family (Figure 1(b)) which has to react with other TFs in order to stimulate or inhibit gene expression [21] in ESCs through heterodimerization with SOX2. It was implicated in embryonic development regulation, cell fate determination, and embryonic stem cell pluripotency [22]. Finally, NANOG (Figure 1(c)) is involved in gene regulation with the aforementioned two transcription factors through their binding to the promoters of several genes which mediates the pluripotency, inhibits embryonic stem cell differentiation, and autorepresses its own expression in differentiating cells [22]. It has been found to be localized mainly in the nuclei of high-grade glioma cells than lower grades [15]. Despite the fact that OCT3/4 and NANOG have shown a direct relationship with the tumor grade, their oncogenic nature in brain tumorigenesis has not been established yet [23].

Up to our knowledge, there were no previous studies that relate the expression of the aforementioned longevity-related transcription factors in GBM patients among the Arab population. Hence, the present study for the first time aimed to quantify the expression levels of these markers in GBM sample of Egyptian patients and to correlate their expressions with the available clincopathological features. A thorough understanding of the relevance of each biomarker in GBM will be in need not only for reliable diagnosis of the disease but also to participate in future drug design for this fetal tumor.

## 2. Materials and Methods

### 2.1. Study Participants and Tissue Samples

The current study included 44 glioblastoma patients (10 females and 34 males, aged 38 to 62 years) assessed retrospectively from archived formalin-fixed paraffin-embedded section (FFPE) specimens of the Pathology Department, Mansoura University Hospitals, Egypt, from 2010 to 2013. They had glioblastoma multiforme grade 4, subjected to surgical removal and postoperative irradiation, and followed up for more than 36 months. Specimens were collected before receiving chemotherapy or radiotherapy prior to surgery. They were compared to 10 FFPE noncancerous brain specimens obtained from patients undergoing brain tissue resection for other reasons collected from the same hospital. Guidelines in the Declaration of Helsinki were followed, and an approval by the Medical Research Ethics Committee of Faculty of Medicine, Suez Canal University, was obtained before taking part. Written informed consent was obtained from all participants before providing the archived tissue samples as part of their routine register in our University Teaching Hospitals.

### 2.2. RNA Extraction

Extraction of total RNA from FFPE specimens was done using RNeasy FFPE Kit (Qiagen, 52304) according to the protocol of the manufacturer. RNA concentration and purity were assessed with NanoDrop ND-1000 spectrophotometer (NanoDrop Tech. Inc., Wilmington, DE, USA), followed by agarose gel electrophoresis (1%) check for RNA integrity.

### 2.3. Reverse Transcription (RT)

Complementary DNA (cDNA) was obtained by total RNA conversion using the High-Capacity cDNA Reverse Transcription Kit (Applied Biosystems, P/N 4368814) with RT random primers on T-Professional Basic, Biometra PCR System (Biometra, Goettingen, Germany), as previously described [12]. Appropriate negative and positive controls were included in each experiment.

### 2.4. Gene Expression Profiling

The Minimum Information for Publication of Quantitative Real-Time PCR Experiments (MIQE) guidelines were followed for the real-time PCR reactions. Pluripotent gene relative expressions were assessed using “Universal PCR master mix II, No UNG (2×)” (TaqMan®, Applied Biosystems, P/N 4440043), TaqMan assay (Applied Biosystems, assay ID Hs02387400_g1 for NANOG, Hs01053049_s1 for SOX2, and Hs03005111_g for OCT3/4) and compared to the endogenous control TATA box binding protein (*TBP*) (Hs00427620_m1) which has been proved in our previous work [24] to be uniformly and stably expressed with no significant difference between GBM and noncancer tissues for gene expression normalization. PCRs were done in 20 *μ*l total volume using “StepOne™ Real-Time PCR System (Applied Biosystems)” as previously described in details [25].

### 2.5. Statistical Analysis

Data analysis was done using PC-ORD ver. 5 software package and Statistical Package for the Social Sciences (SPSS) for windows software (version 22.0). Two-tailed statistical tests were used for continuous and categorical variables. Correlation analysis between the variables was performed via Pearson's correlation coefficient. *p* value < 0.05 was considered significant. The fold change of mRNA expression in each patient cancer tissue relative to the mean of controls was calculated using Livak method that depends on the quantitation cycle (*C*_q_) value with the following equation: relative quantity = 2^−ΔΔ*C*_q_^ [26]. The diagnostic performance of pluripotent genes was evaluated by receiver operating characteristic (ROC) analysis. Kaplan–Meier estimator was generated for survival analysis, and log-rank test was applied for different Kaplan–Meier curve (stratified by clinicopathological features) comparisons. Linear regression analysis using ENTER method was performed to evaluate potential factors affecting the overall survival of patients. Two-way Hierarchical cluster analysis was run for exploratory multivariate analysis. Ward's method and Euclidean (Pythagorean) were adjusted for linkage method and distance measure, respectively, with a beta value of −0.75 to reach the minimum % of chaining. Principal Component Ordination analysis was used to visualize clustering of patients according to their clinicopathological characteristics [27].

## 3. Results

### 3.1. Expression Profile of Pluripotent Genes

**Figure 2:**
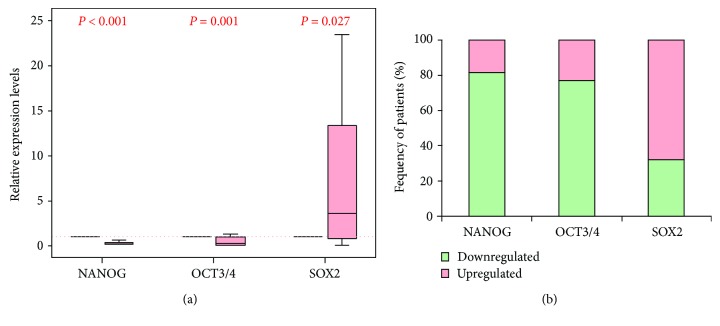
Expression profile of pluripotent genes in GBM patients compared to controls. (a) Values are presented as medians and quartiles of fold change relative to controls. The box defines upper and lower quartiles (25% and 75%, resp.) and the Whisker bars indicate upper and lower adjacent limits. *TBP* was used as an internal control. Noncancer tissues was set to have a relative expression value of 1.0. Mann–Whitney *U* test was used for comparison. *p* value < 0.05 was considered statistically significant. (b) Frequency of patients with up- and downregulated genes.

**Figure 3:**
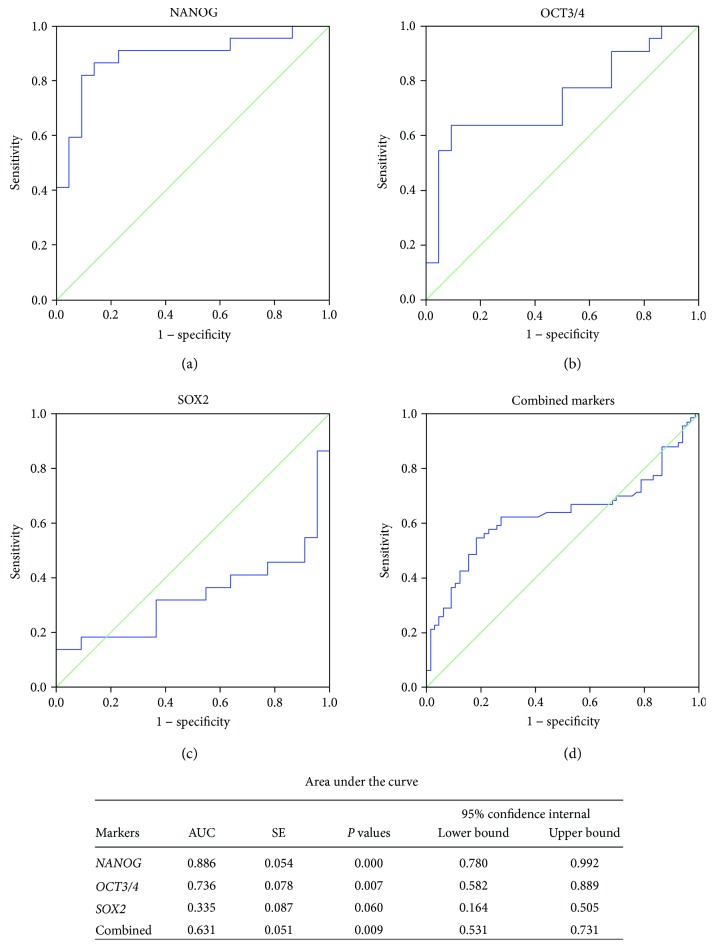
Diagnostic performance of pluripotent genes to discriminate between GBM and noncancer samples. NANOG and OCT3/4 showed high diagnostic values as biomarkers for GBM.

Baseline clinical features of the study participants are illustrated in Table 1. Relative expression analyses of pluripotent genes in brain cancer specimens were compared to *TBP*. Our results revealed that the expression levels of *NANOG* and *OCT3/4* were significantly downregulated (*p* < 0.001 and = 0.001, resp.) while that of *SOX2* was significantly upregulated (*p* = 0.0027) in tumor specimens compared to noncancer tissues (Figures 2(a) and 2(b)). Both NANOG and OCT3/4 mRNAs showed high diagnostic values as biomarkers for GBM (AUC = 0.886 ± 0.054 and 0.736 ± 0.078, resp.) (Figure 3).

### 3.2. Association with Clinicopathological Characteristics and Survival Analysis

**Figure 4:**
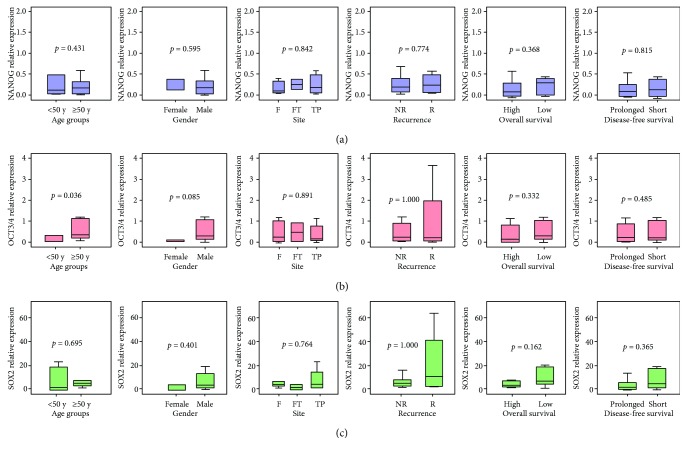
Association between gene expression and the clinicopathological features. Values are presented as medians and quartiles of fold change relative to controls. The box defines upper and lower quartiles (25% and 75%, resp.) and the whisker bars indicate upper and lower adjacent limits. TBP was used as an internal control. Noncancer tissues were set to have a relative expression value of 1.0. Mann–Whitney *U* and Kruskal-Wallis tests were used for comparison. *p* value < 0.05 was considered statistically significant. F: frontal tumor site; FT: frontotemporal; TP: temporoparietal; R: recurrent; NR: nonrecurrent.

**Figure 5:**
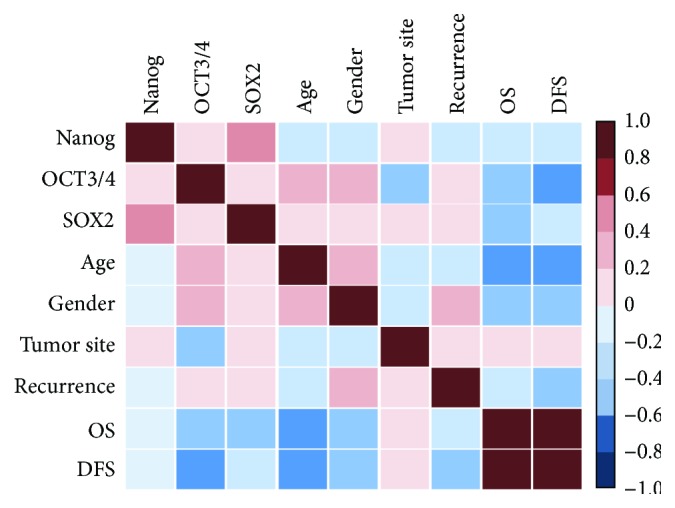
Correlation matrix between transcriptomic signature and the clinicopathological features. Pearson's correlation analysis was performed and represented as color gradient.

Higher *OCT3/4* gene expression was noted in elder GBM patients (*p* = 0.036). No statistically significant association was found with any other parameters (Figure 4). Correlation analysis revealed moderate correlation between *NANOG* and *SOX2* gene expression profile (*r* = 0.484, *p* = 0.023). In addition, elder age of patients was associated with poor overall survival (OS) (*r* = −0.479, *p* = 0.024) and disease-free survival (DFS) (*r* = −0.481, *p* = 0.023) (Figure 5).

**Figure 6:**
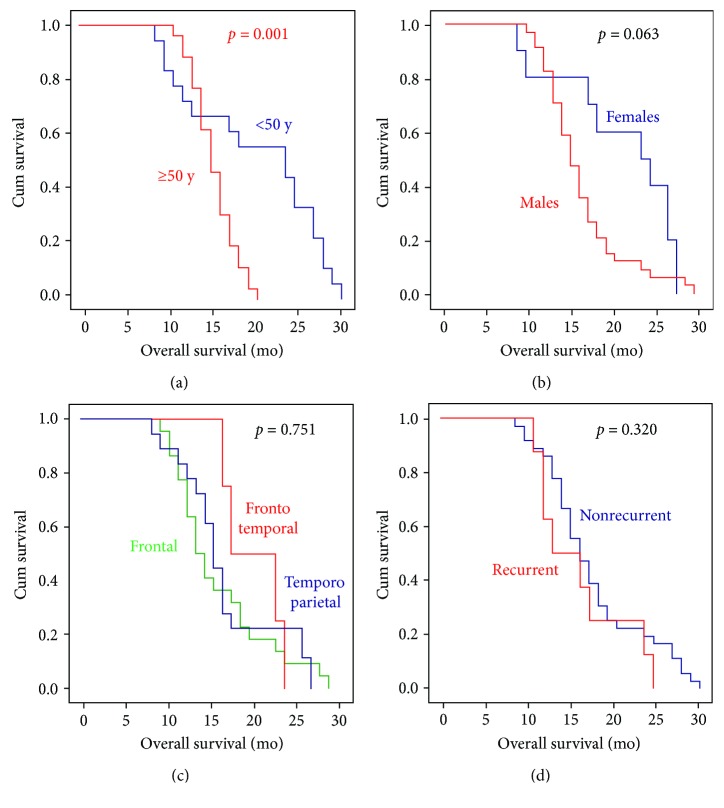
Kaplan–Meier survival curve in GBM patients. Log-rank (Mantel-Cox) test was used for comparison. Statistical significance at *p* < 0.05.

Linear regression analysis was performed to evaluate potential factors affecting overall survival of patients. None of the genes or clinicopathological variables was determined as a good prognostic marker for patients' survival in the study population (Table 2). However, survival analysis in GBM by log-rank and Tarone-Ware tests showed poor OS among elder patients (Figure 6 and Table S1).

### 3.3. Multivariate Analysis

**Figure 7:**
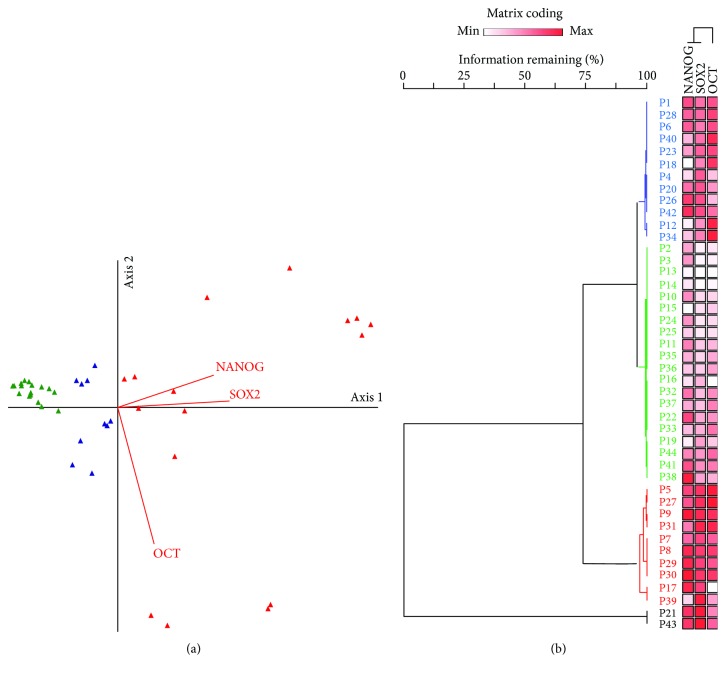
Multivariate analyses cluster GBM patients according to transcriptomic signature. PC-ORD v5.0 was used for exploratory multivariate analysis. Data set was profiled by the program. There was no need for transformation as beta diversity was zero and there was no outlier. (a) Ordination graph by PCO and (b) two-way hierarchical cluster analysis. The following parameters were adjusted: linkage method; Ward's method; distance method; Euclidean method; relativizing matrix by column maximum; and matrix coding percentile by column. Percent chaining = 7.38. Clustering identified three patient groups according to their gene expression. The red clade for overexpression of the three pluripotent genes, the green clade discriminates patients with gene downregulation, and the blue clade has variable degrees of expression. Two samples (black clade) were out-group from the other clusters.

Exploratory multivariate analysis by principle component and hierarchical cluster analyses classified patients into 3 groups based on the relative expression of the combined genes (Figure 7). However, there was no clear demarcation found between patients according to age, gender, tumor site, and recurrence (Figure S1).

## 4. Discussion

The presence of a significant heterogeneity in certain types of solid tumors including GBM is becoming obvious. Hence, it will be rational to search for and evaluate specific molecular markers that could assist in diagnosis and/or prognosis of these tumors and could act as targeted molecular markers for personalized therapy [7]. Here, we attempted to investigate the presence of a molecular signature of longevity-related genes (*SOX2*, *NANOG*, and *OCT3/4*) by examining their mRNA expression in GBM tissues relative to noncancer tissues. Our analyses revealed that the expression level of SOX2 was significantly upregulated. This finding was consistent with several independent cohorts [28–30] and in part with Guo et al., [13] who detected an overexpression of SOX2 mRNA in grade IV gliomas compared to grade II. Of the three longevity-related factors, SOX2 seems to be the playmaker in the development of brain tumors [18]. When overexpressed, it promotes cell cycle progression into S phase and proliferation [3, 20, 28, 31], which were attenuated by application of SOX2-RNAi (RNA interference) therapy [32]. At the cellular level, Garros-Regulez et al. [33] proposed SOX2 upregulation via activation of GBM-specific signaling pathways that maintain the overexpression of SOX2 via transforming growth factor-beta (TGF-*β*), Sonic Hedgehog (SHH), epidermal growth factor receptor (EGFR), and fibroblast growth factor receptor (FGFR) pathways. In addition, *SOX2* gene amplification and DNA promoter hypomethylation have been reported in a group of GBM patients to expand the mechanism responsible for SOX2 upregulation [34].

Despite that our in silico analysis revealed that the expression of the studied stem-related factors has similar colocalization and physical interactions with each other [12], they seem to be differentially expressed independently in the current samples. We found that *NANOG* and *OCT3/4* were significantly downregulated in GBM tissues. Our finding might seem contradictory to the stemness role these pluripotent transcription factors play; however, it is worth to emphasize that the mechanistic functions of SOX2, OCT4, and NANOG in cancer cells are a little different in each stage of tumor progress. Kallas et al. reported high levels of SOX2, OCT4, and NANOG transcription factor expressions at the beginning of their tested human embryonic stem cell differentiation. However, on progress of the differentiation process, a decline in OCT4 and NANOG expression levels was observed, while expression of SOX2 was kept at a high level [35]. They suggested that the pluripotency is maintained by a transcriptional network that is harmonized by the aforementioned core transcription factors. During differentiation, the epigenetic modifications could play a role in level modulation of these factors.

The other possible reasons for inconsistency of gene expression for the three stem cell marker studies could be sampling bias and/or relatively low expression levels of these factors within the individual GBM tissue examined in the current study [36]. This could be explained by the unique stem cell signature that has been implied by each tumor due to the inherent intratumor heterogeneity within GBM tissues [37–39]. Our multivariate analysis and the hierarchical cluster analysis confirmed the previous suggestions by revealing classification of the study population into 3 groups based on the combined gene expression that confirm a specific protumorigenic profile. Similar to other combinations of cancer stem cell markers in other types of cancer [40, 41], previous studies revealed that cancer stem cells which were isolated using different markers in the same cancer phenotype had different expression profiles quantified by real-time PCR. Combined expression analysis might more accurately identify true cancer stem cells for each type of cancer [40], including GBM tumors.

Ji et al. reported that unlike normal stem cells, OCT4 could be dispensable for self-renewal, survival, and differentiation of transformed cells. They provided direct evidence for the functional divergence of OCT4 from the pluripotent state following the cancer tissue transformation [42]. This could support the downregulation of this stem-related marker noted in the current advanced stages of GBM cases. Additionally, Bradshaw et al. [36] reported low OCT4 relative expressions at the transcription and protein levels within their FFPE GBM samples. They speculated that the relatively OCT4-expressing cell low number could indicate the most primitive stem cell population within GBM which may possibly bring about the rest of downstream cells within the GBM tumor. Otherwise, the SOX2 ubiquitous redundancy is more likely to be expressed in the more differentiated cells reflecting its usefulness as a potential progenitor cell marker within the GBM tissues [36].

In contrast to the finding of Zbinden et al. [43] that NANOG was essential for GBM tumourigenicity in orthotopic xenografts, we found downregulation of this marker in the current GBM samples. We speculated that this difference could be due to either the low NANOG-expressing cell number within the study samples as mentioned above for the OCT4 marker or the type of NANOG transcript that has been quantified by the available quantitative PCR analysis at the time of the current work which preferentially recognized the varying levels of NANOG expression. As NANOG is coded by two genes (i.e., *NANOG* and *NANOGP8*) in human, it has been found that *NANOGP8* is the most abundantly expressed of the two NANOG-encoding genes in GBMs, accounting for more than ninety percent of all NANOG-encoding mRNAs in a number of previously tested cases [43]. However, future lineage analyses will be required for unravelling the high NANOG-expressing cell nature and NANOG expression stability as recommended by the latter researchers.

Correlating the available clincopathological features including the survival data of GBM cases with the gene expression results revealed that poor overall survival and disease-free survival were found significantly among patients as reported by previous studies [44, 45]. Despite that GBM can occur in individuals of any age according to the previous population-based studies, the median age is nearly above 60 years. Additionally, primary GBMs have been reported to develop commonly in older individuals (mean, 55 years), whereas secondary ones were found in middle-aged subjects (39 year olds) [4].

## 5. Conclusion

The current study findings highlighted the dysregulated longevity-related gene expression in GBM Egyptian cases that could have a potential role in carcinogenesis and procuration of stemness-like properties in this type of tumors. The current study could be limited by the relatively small sample size and the fact that all patients have grade IV gliomas, although this last issue increases the specificity of the study results that confined to one stage of GBM. Additional large-scale studies including different glioma grades are recommended to evaluate the relation of the studied longevity-related gene expression with different WHO grades as well as to confirm their putative role as diagnostic and/or prognostic biomarkers. These could be an interesting era for future individualized molecular-targeted therapy for GBM patients.

## Figures and Tables

**Table 1:** Characteristics of GBM patients.

Variables	Number (%) or mean ± SE
*Age*	
Mean ± SE	51.4 ± 0.97
Age categories	
35–50 y	18 (40.9)
>50 y	26 (59.1)
*Gender*	
Female	10 (22.7)
Male	34 (77.3)
*Tumor site*	
Frontal	22 (50)
Frontotemporal	4 (9.1)
Temporoparietal	18 (40.9)
*Recurrence*	
Nonrecurrent	36 (81.8)
Recurrent	8 (18.2)
*Disease-free survival* (*months*)	
Mean ± SE	15.1 ± 0.85
Range	6–27
Prolonged DFS (>1 y)	28 (63.6)
Short DFS (≤1 y)	16 (36.4)
*Overall survival (months)*	
Mean ± SE	15.6 ± 0.86
Range	8–27
High survival (>1 y)	30 (68.2)
Low survival (≤1 y)	14 (31.8)

**Table 2:** Linear regression analysis to determine predictors for survival.

	Unstandardized coefficients		Standardized coefficients			95% confidence interval for *B*	
	*B*	Std. error	Beta	*t*	Sig.	Lower bound	Upper bound
(Constant)	34.675	10.593		3.273	0.006	11.955	57.395
Age	−0.318	0.220	−0.363	−1.448	0.170	−0.790	0.153
Gender	−2.142	3.510	−0.170	−0.610	0.551	−9.671	5.387
Tumor site	0.079	1.498	0.014	0.053	0.959	−3.134	3.292
Recurrence	0.501	3.635	0.036	0.138	0.892	−7.295	8.296
NANOG	0.374	3.095	0.033	0.121	0.905	−6.263	7.011
OCT3/4	−0.943	1.339	−0.173	−0.705	0.493	−3.815	1.928
SOX2	−0.085	0.123	−0.214	−0.691	0.501	−0.348	0.178

## Abbreviations

*C*_q_: Quantitation cycle
DFS: Disease-free survival
ESCs: Embryonic stem cells
GBM: Glioblastoma multiforme
MIQE: Minimum Information for Publication of Quantitative Real-Time PCR Experiments
NANOG: Nanog homeobox
OCT: Octamer-binding transcription factor
OS: Overall survival
ROC: Receiver-operating characteristic
RT: Reverse transcription
SOX2: Sex-determining region Y-Box.

## Supplementary Materials

Supplementary Materials Table S1: Kaplan–Meier curves comparing survival of patients with different clinical variables. Figure S1: multivariate analysis stratified by clinicopathological features.
