# Supplementary material for: Longevity-Related Gene Transcriptomic Signature in Glioblastoma Multiforme
Source: Oxid Med Cell Longev. 2018 Mar 21;2018:8753063. doi: 10.1155/2018/8753063 (PMC5914091; doi:10.1155/2018/8753063)
Supplement: Supplementary Materials — Table S1: Kaplan–Meier curves comparing survival of patients with different clinical variables. Figure S1: multivariate analysis stratified by clinicopathological features. [file 8753063.f1.docx]

| **Supplementary Table S1. Kaplan-Meier curves comparing survival of patients with different clinical variables.** | | | | | | | | |
| --- | --- | --- | --- | --- | --- | --- | --- | --- |
|  |  |  |  | 95% CI | | Overall comparisons | | |
|  |  | Median | SE | Lower border | Upper border | Log Rank | Breslow | Tarone-Ware |
| Age | <50 y | 22 | 5.270 | 11.670 | 32.330 | **0.001** | 0.070 | **0.012** |
|  | ≥50 y | 14 | 0.635 | 12.754 | 15.246 |  |  |  |
| Gender | Female | 22 | 4.743 | 12.703 | 31.297 | 0.063 | 0.056 | 0.039 |
|  | Male | 14 | 0.728 | 12.574 | 15.426 |  |  |  |
| Tumor site | F | 13 | 0.938 | 11.161 | 14.839 | 0.751 | 0.294 | 0.447 |
|  | FT | 17 | 3.000 | 11.120 | 22.880 |  |  |  |
|  | TP | 15 | 0.703 | 13.623 | 16.377 |  |  |  |
| Recurrence | NR | 15 | 0.998 | 13.043 | 16.957 | 0.320 | 0.354 | 0.346 |
|  | R | 12 | 2.828 | 6.456 | 17.544 |  |  |  |
| F, frontal tumor site; FT, fronto-temporal; TP, tempero-parietal; R, recurrent; NR, non-recurrent. Bold data indicate significance at *p* < 0.05. | | | | | | | | |


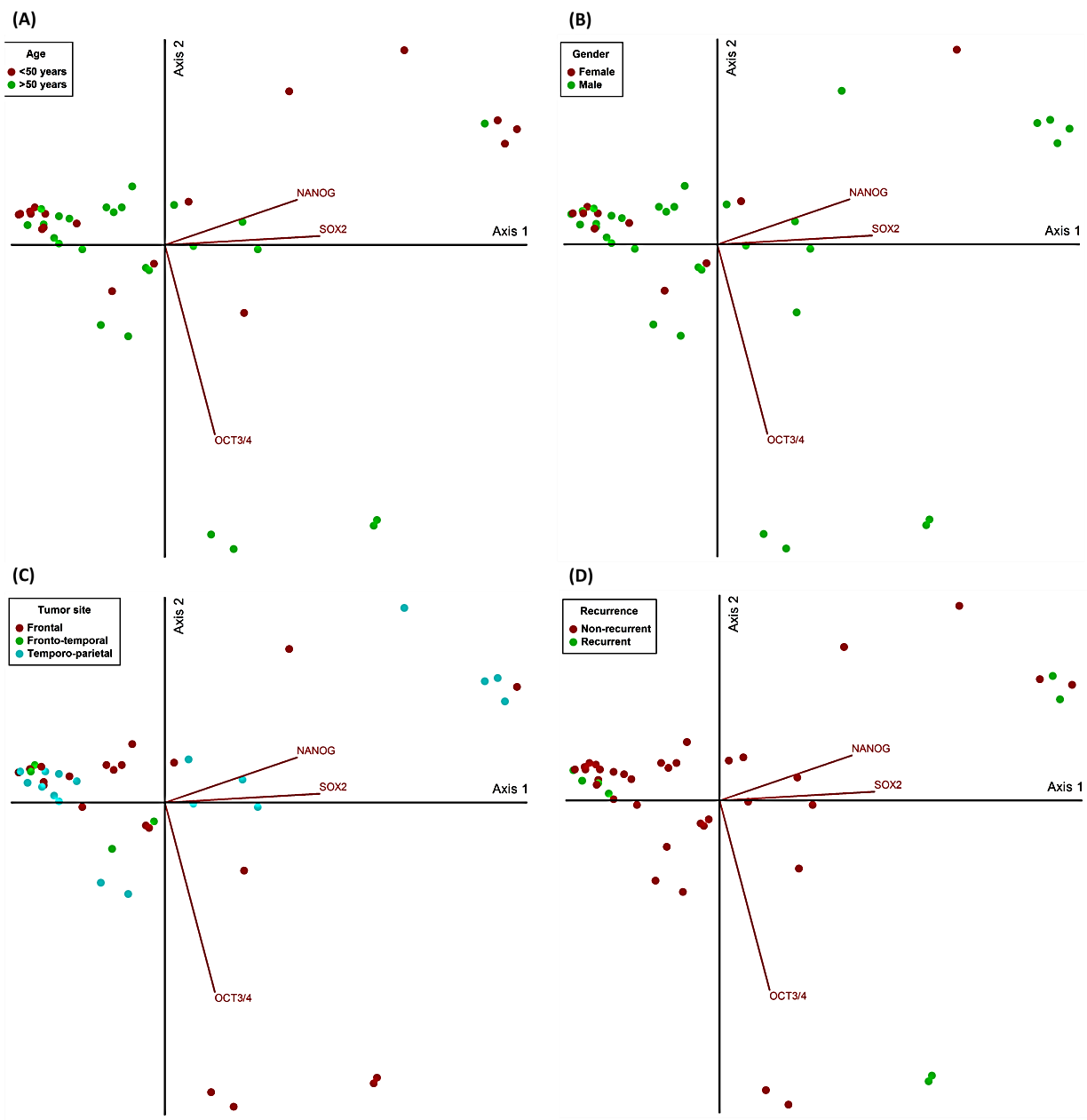


**Supplementary Figure 1. Multivariate analysis stratified by the clinico-pathological features.** Principal Component Analysis scored by distance-based biplot. Axis 1, 2 and 3 identified 51.03%, 31.76% and 17.20% of variance, respectively. No clear demarcation by gene expression signature between patients according to age, gender, tumor site and recurrence.
